# Berbamine and thymoquinone exert protective effects against immune-mediated liver injury *via* NF-κB dependent pathway

**DOI:** 10.3389/fvets.2022.960981

**Published:** 2022-07-26

**Authors:** Sarmed H. Kathem, Waleed K. Abdulsahib, Munaf H. Zalzala

**Affiliations:** ^1^Department of Pharmacology and Toxicology, College of Pharmacy, University of Baghdad, Baghdad, Iraq; ^2^Department of Pharmacology and Toxicology, College of Pharmacy, Al Farahidi University, Baghdad, Iraq

**Keywords:** berbamine, thymoquinone, hepatitis, Concanavalin A, acute liver injury, TNF-α, IFN-γ, NF-κB

## Abstract

**Background:**

Immune-mediated hepatitis is a severe impendence to human health, and no effective treatment is currently available. Therefore, new, safe, low-cost therapies are desperately required. Berbamine (BE), a natural substance obtained primarily from *Berberis vulgaris* L, is a traditional herbal medicine with several bioactivities, such as antimicrobial and anticancer activities. Thymoquinone (TQ), a phytochemical molecule derived from the *Nigella sativa* plant's black cumin seeds, has attracted interest owing to itsanti-inflammatory, antioxidant, and anticancer properties.

**Aim:**

This current study's aims was to examine the protective impacts of BE and TQ in Concanavalin A (ConA)- induced acute liver injury and the action's underlying mechanism.

**Methods:**

sixty mice of both sexes were used and divided into four groups (each group with six mice) as follows: Group I obtained distilled water (negative control group). Group II received distilled water with a single dose of 0.1 ml ConA (20 mg/kg) on day 4 by retro-orbital route (model group). Groups III and IV received BE (30 mg/kg/day) and TQ (25 mg/kg/day), respectively, by oral gavage for four successive days, with a single dose of ConA (20 mg/kg) on day 4, then all animals were sacrificed after 8 h and prepared for liver and blood collection.

**Results:**

ConA administration increased the ALT, AST, TNF-α, INFγ, and NF-κB significantly (*p* < 0.001) in the model group. Both BE and TQ could reduce these parameters significantly (*p* < 0.001) in groups III and IV, respectively, compared to the model group.

**Conclusion:**

Both BE and TQ prominently attenuated ConA immune-mediated liver injury. These findings give a remarkable insight into developing a new therapeutic agent for treating hepatitis and other autoimmune diseases.

## Introduction

Hepatitis can be induced by several elements, involving alcohol, viruses, medications, damaging immunological substances, and idiopathic variables, and it is still a serious public health concern (1). Hepatitis symptoms include the inflammatory cytokines' release, a rise in alanine aminotransferase (ALT) and aspartate aminotransferase (AST), and hepatocyte death and necrosis (2). Even though numerous treatments are currently employed in clinics, the therapeutic outcome is not optimal. As a result, effective therapeutic options must be investigated (3). Tiegs et al. first employed ConA, a plant agglutinin isolated from Brazilian rubber beans, to investigate liver injury (4). It is often utilized to induce an experimental model of acute liver injury. This agent causes severe liver inflammation, abnormally high serum transaminases, tissue necrosis, and potential organ failure (5). Lymphocyte cells of type T and natural killer T (NKT) cells are mainly recruited and activated by ConA (3). Even though ConA is relatively deleterious to hepatic cells *in vitro*, most *in vivo* investigations show that lymphocytes influence the creation and progression of the liver injury model (6). The hepatic cell damage after a ConA challenge has been linked to several mechanisms. The injection of agents that interfere with TNF-α can protect the liver from the damage caused by ConA (7).

In addition, recombinant interleukin-6, neutralized with TNF-α, prevents liver cell destruction in acute ConA hepatitis (8). TNF-α can trigger hepatocyte death directly *via* TNF-receptor signaling, which activates caspase-8, culminating in the activation of caspase-3 and mitochondrial cytochrome c (9). Apoptosis is affected by caspase cascades, which cause the cleavage or stimulation of molecules involved in cell death. Proteins block caspase cascades at various stages and can mediate cell survival pathways (10).

In contrast, TNF signaling can trigger alternate pathways in hepatocytes, stimulating downstream signals, including NF-κBκκ and NF-κBκκ essential modulators (NEMO), which have been demonstrated to suppress apoptosis by suppressing c-Jun N-terminal kinase (JNK) activation and inducing Bcl-XL (11). As a result, TNF-α can only cause liver cellcell deterioration cellif NF-κBκκ activation is blocked sufficiently. Therefore, an elevated expression of NF-κB has been observed κκ in ConA-induced acute hepatic injury to counteract the increased apoptosis and fragmentation of DNA. Moreover, apoptotic body production has been observed in hepatocytes as early as 5 h after ConA administration, much before increased serum transaminase levels, clearly indicating programmed cell death processes (3). Other signals, involving Fas ligand or TNF-related apoptosis-inducing ligand (TRAIL), have been linked to hepatocyte damage in the ConA animal model and are efficient in triggering the death of hepatocyte cells *via* receptor-interacting protein 1 (RIP)1/RIP3 kinase signaling and TRAIL-induced necroptosis (12). Although necroptosis shares some characteristics of regulated cellular deterioration, including nuclear fragmentation and mitochondrial potential breakdown, it finally leads to abnormal cell degradation, leading to the release of cytoplasmic components and thus activating immune reactions. In addition, the cytokine interferon (IFN)-γ activated by T-cell is required for ConA production of liver inflammation (13). The CD8 cytotoxic T-lymphocyte (CTL) involvement in hepatocyte apoptosis has been debated (13). However, at least one study found that CTL significantly influences perforin-stimulated hepatic cell apoptosis (14). After intravenous administration, ConA largely accumulates in the liver, where it binds to liver sinusoidal endothelial cells that line the tiny hepatic blood capillaries. As a result, it can operate as a bridging component, causing passenger T-lymphocytes to enter a state of firm cellular arrest, which leads to their activation. Knock-out mice for P-selectin or adhesion molecules involving lymphocyte function-correlated antigen (LFA)-1 (15) are protected from ConA liver injury, supporting this theory.

Berbamine (BE), a natural substance obtained primarily from *Berberis vulgaris* L, is a traditional herbal compound with several bioactivities (16), such as antimicrobial and anticancer characteristics (17). BE has previously been shown to increase TNF-α-induced protein at three levels, decrease IκB kinase (IKK) phosphorylation, block p65 nuclear translocation, and act as a nuclear factor kappa B (NF-κB) inhibitor (18). Additionally, BE has an immunomodulatory effect and is reported to reduce signal transducer and activator of transcription 4 (STAT4) and IFN-γ expression, specifically in experimental autoimmune encephalomyelitis (19). BE has lately shown promise as a treatment for reducing inflammation caused by various malignancies, hence preventing tumor cell invasion (18).

Thymoquinone (TQ), a phytochemical product derived from the Nigella sativa herb's black cumin seeds (20), has attracted interest attributable to its anti-inflammatory, analgesic, antipyretic, antioxidant, and anticancer properties (21). Pro-inflammatory cytokines, elastase, myeloperoxidase, lipoxygenase (LOX) and cyclooxygenase (COX) enzymes, reactive oxygen species (ROS), and epigenetic alterations can all be inhibited by this compound (21). It inhibits various signaling pathways, including Janus kinase/signal transduction (NF- κβ), stimulator of mitogen-activated protein kinase (MAPK), and transcription (JAK-STAT). Therefore, it can improve autoimmune disease therapies (22). A previous study conducted by our research team demonstrates a good protective effect of TQ (15 mg and 30 mg/ kg) against immune-mediated liver injury (23). As a result, we considered in the current study the protective impacts of BE and TQ and the action's main mechanism in the ConA-triggered model of autoimmune hepatitis. We expected that BE and TQ possibly would lower TNF-α, IFN-γ, and NF- κBκκ levels and improve liver function as evaluated by measuring the level of hepatic enzymes.

## Methods

### Animals

To evaluate the effect of BE and TQ, mice weighing about 25–30 g (7–9 weeks old) were used. Mice were kept in a clean room under controlled temperature and humidity with a 12-h light/12-h dark cycle each day. Mice also had unrestricted access to water and food. All experiments were performed accom the health guidelines of the institute's care and use of laboratory animals. The Scientific and Ethical Committee approved the experimental protocol at the College of Pharmacy, University of Baghdad. Additionally, the study followed the recommendations in the ARRIVE guidelines.

### Experimental design

**Figure 1:**
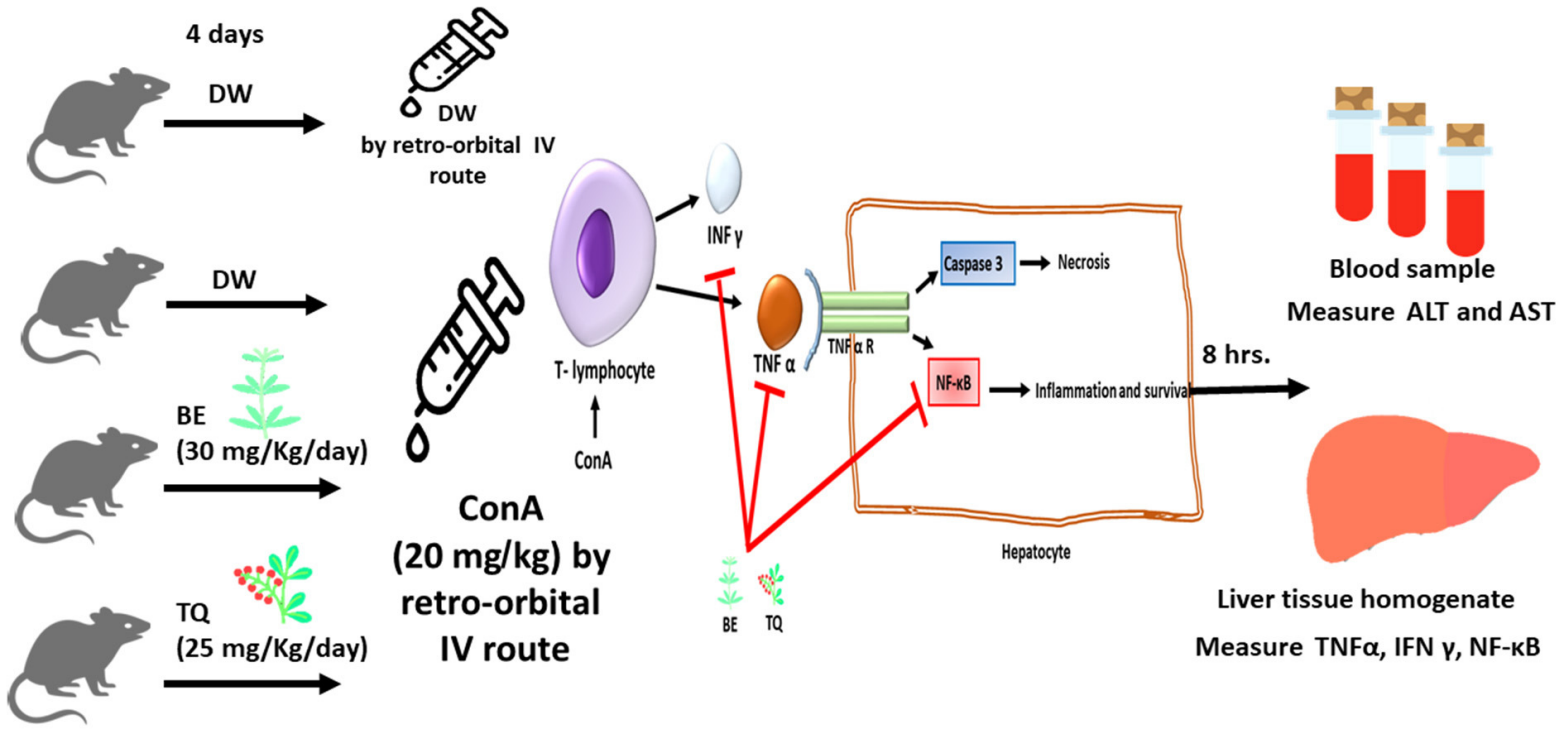
The schematic diagram of the study design with the suggested mechanism of action of Berbamine and Thymoquinone.

Twenty-four male mice were utilized in the study to achieve its goals. Four groups of six mice each were created at random from the mice, as shown in: group I: starting on day 1, animals in this group obtained 0.1 ml of distilled water orally every day for 4 days. On day 4, 0.1 ml of normal saline was administered retro-orbitally, and the animals were sacrificed 8 h later. This group performed the role of a negative control group. Group II: starting on day 1, animals in this group obtained 0.1 ml of distilled water orally daily for 4 days. On day 4, 0.1 ml of ConA (20 mg/kg) was administered *via* retro-orbital IV, and the animals were sacrificed 8 h later. This group described as a model of immune-mediated liver failure (model group). For 4 days straight starting on day 1, group III obtained 0.1 ml be dissolved in DMSO (30 mg/Kgkgkg) by oral gavage. On day 4, they also got a single dose of cona (20 mg/kg), after which they were sacrificed after 8 h. In accordance with Figure 1, group IV was given 0.1 ml TQ (25 mg/kg dissolved in corn oil) by oral gavage over the course of 4 days starting on day 1, along with a single dose of ConA (20 mg/kg), and was then sacrificed after 8 h. After Cervical dislocation and an intraperitoneal injection of pentobarbital sodium (150 mg/kg) to cause euthanasia, all animals were then ready for liver and blood gathering.

### Reagents and chemicals

The lyophilized ConA, berbamine dihydrochloride, and thymoquinone powder with more than 98% purity were purchased from Hangzhou Hyper chemicals (China). DMSO, corn oil, and phosphate buffer used to dissolve the tested agents were purchased from Sigma Aldrich (Germany). The AST and ALT activity kit tests were purchased from Randox laboratories (United Kingdom). Mouse INF-γ and TNF-α -ELISA kits were provided from Beijing Solarbio Science (China), NF-κBκκ and YWHAZ primers were purchased from Macrogen (Korea), and Mic qPCR Cycler was used from BioMolecular System (Australia).

### ConA preparation

Each vial containing 100 mg of lyophilized ConA powder was used to prepare a stock solution in 20 ml phosphate buffer saline. The 20 ml stock solution was divided into 14 tubes, each containing 1.4 ml was frozen at −20 °C to be used later. The animals were anesthetized with diethyl ether and received 20 mg/kg of ConA solution intravenously *via* the retro-orbital route (3).

### Blood collection

An approximate 1.5 ml of blood was taken from the retro-orbital region of mice on day 5 and precisely 8 h after ConA injection. In order to gather serum, blood was left at room temperature for 30 min. It was centrifuged in a cold centrifuge at 4 °C for 15 min. at 5,000 rpm. For the purposes of determining aspartate transaminase (AST) and alanine aminotransferase (ALT), the serum was gathered and frozen at −20 °C (24).

### Preparation of liver tissue homogenate

Each mouse's liver was quickly removed and rinseed in ice-cold normal saline following euthanasia and dissection. A 1.5 ml Eppendorf tube containing phosphate buffer saline was then filled with 150 mg of liver tissues. An electrical homogenizer was utilized to blend the liver tissues together. The homogenate was then centrifuged in a cold centrifuge for 15 min at 5,000 rpm. Then, the supernatant fluid was gathered and frozen for further utilization to evaluate TNF α and INF γ. Determination of serum ALT and AST was performed by colorimetric assay according to the manufacturer protocol. TNF-α and INF-γ were quantified using ELISA kits according to manufacturer protocol (25).

### Determination of tissue NF-κB gene expression

Analysis of gene expression level of NF-κB genes depended on the mRNA concentration after conversion to cDNA. The processes included total RNA extraction and purification, real-time quantitative polymerase chain reaction (RT-qPCR) amplification, and data analysis. YWHAZ (Tyrosine 3-Monooxygenase/Tryptophan 5-Monooxygenase Activation Protein Zeta) was utilized as the housekeeping control gene (26). The sequence of primers utilized in the study is shown in Table 1.

**Table 1:** The primer sequence used to analyze NF-κB gene expression.

**Primer**	**Sequence**
NF-κB1-F	5^′^-AAGACAAGGAGCAGGACATG-3^′^
NF-κB1-R	5^′^-AGCAACATCTTCACATCCCC-3^′^
YWHAZ-F	5^′^-GATGAAGCCATTGCTGAACTTG-3^′^
YWHAZ-R	5^′^-GTCTCCTTGGGTATCCGATGTC-3^′^

### Statistical analysis

The study's numerical data were expressed as mean ±standard error of the mean (SEM). The Statistical Package of Social Science (SPSS) software version 25 was used for all statistical analyses. Comparisons between groups were made utilizing (ANOVA and Student t-tests). At (*P*-value <0.05), differences were deemed significant (27).

## Results

### Effect of BE and thymoquinone on liver enzymes

**Figure 2:**
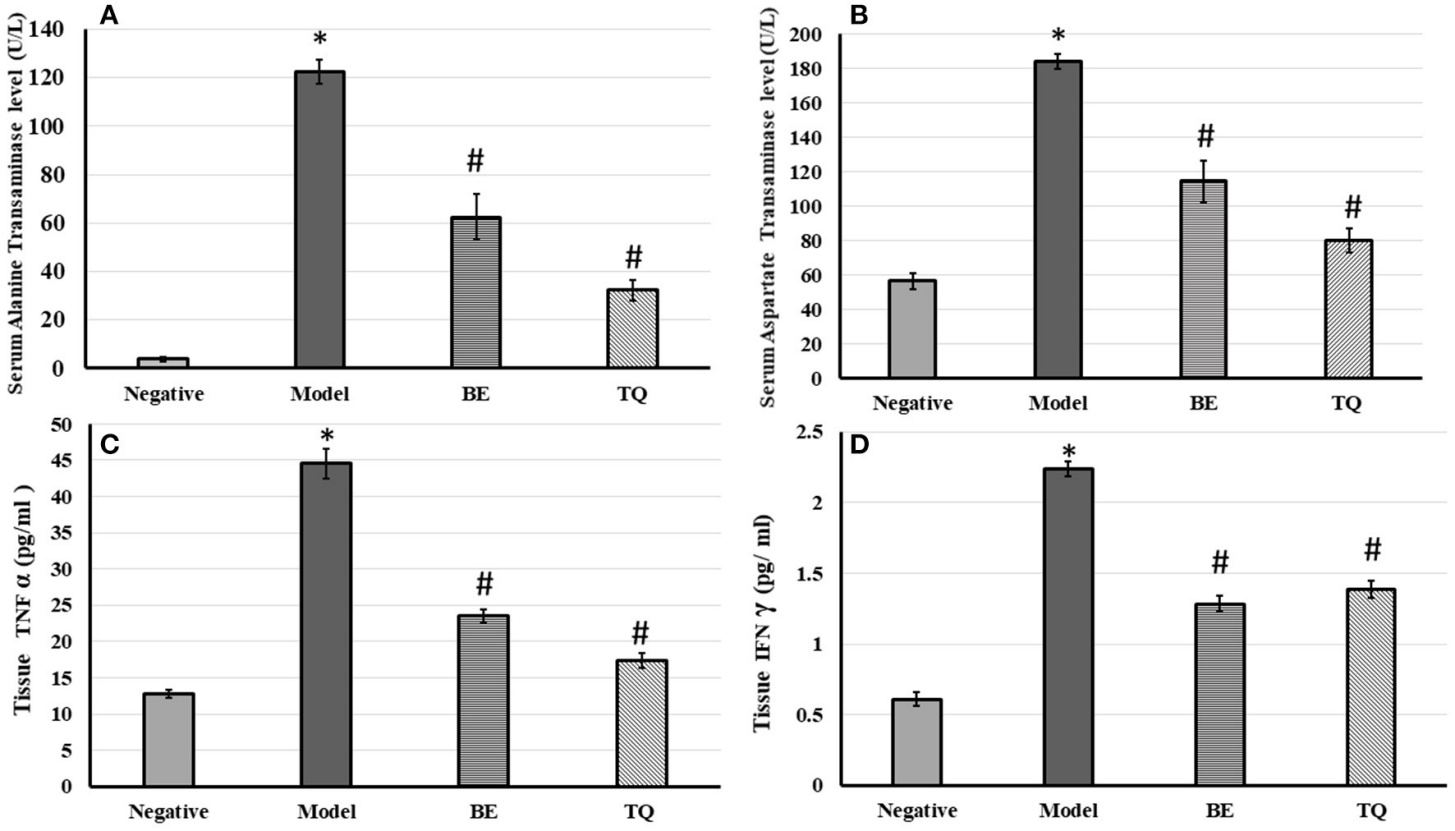
The effects of BE (30 mg/kg/day) and TQ (25 mg/kg/day) on **(A)** serum ALT level; **(B)** AST level; **(C)** tissue TNF α expression and **(D)** tissue IFN-γ expression. * Denotes a significant difference compared to the negative group (p< 0.001). # Denotes a0101 significant difference compared to the model group (*p*< 0.0010101). BE, Berbamine; TQ, Thymoquinone (*n* = 6).

After the injection of ConA, the ALT significantly (*p* < 0.001) increased from (3.87 ± 1.05) in the negative group to (122.41± 4.85) in the model group. Also, the AST significantly increased (*p* < 0.001) from 56.41± 4.56 to 184.24 ± 4.19, as shown in Figures 2A,B. Additionally, contrasted to the model group, there was, additionally a significant decline in ALT and AST levels (*p* < 0.001) after 4 days of BE administration (group III).

Pretreatment with TQ also significantly reduced ALT and AST levels (*p* < 0.001) contrasted to the model group. Both BE, and TQ significantly reduced TNF-α (*p* < 0.001) contrasted to the induced group, as shown in Figure 2C. In addition, the TQ group had a stronger regulatory effect on this cytokine than the BE group (*p* < 0.05).

**Figure 3:**
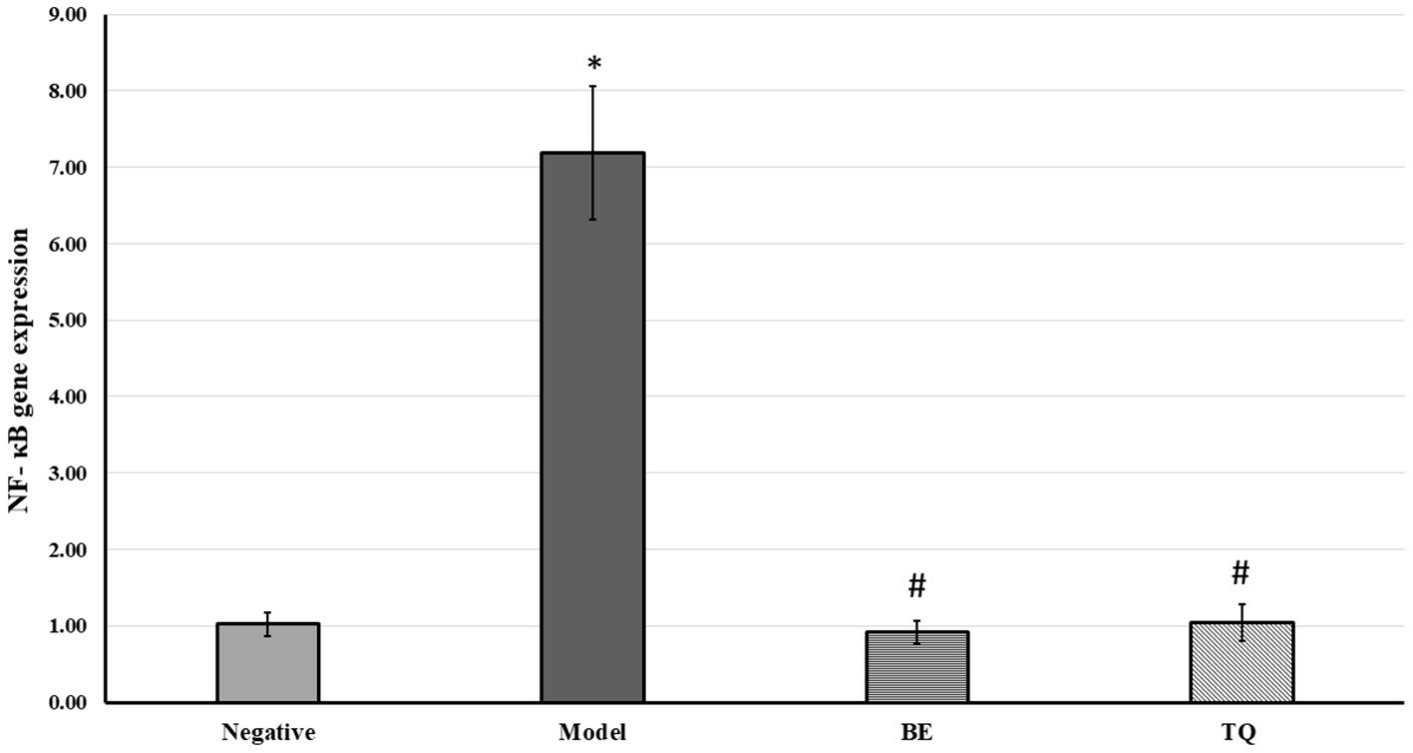
The effects of BE (30 mg/kg/day) and TQ (25 mg/kg/day) on tissue NF-κB gene expression. * Denotes a significant difference compared to the negative group (*p*< 0.001). # Denotes a0101 significant difference compared to the model group (*p*< 0.0010101). BE, Berbamine; TQ, Thymoquinone (*n* = 6).

Figure 2D demonstrateded a significant (p < 0.001) decline in the tissue expression of IFN- γ in groups IIIgroupsgroups and IV contrasted to group II. Finally, both the tested agents reduced significantly (*p* < 0.001) the gene expression of NF-κB compared to the acute liver injury group, as shown in Figure 3.

## Discussion

Immune-mediated hepatitis is an intense impendenceto human health, and no effective treatment is currently available. As a result, new, safe, and low-cost therapies are desperately needed. In this study, we demonstrated that BE and TQ had a protective impact on ConA-induced acute liver injury in mice, indicating a novel role for herbal medicine in treating autoimmune diseases (AID). We observed that pretreatment with BE or TQ for 4 days reduced the ALT and AST levels, indicating that these agents played an important role in reducing liver cell necrosis. These findings were consistent with those of LIU Xin-Yu et al., who demonstrated the ability of BE to suppress both AST and ALT in ethanol-induced liver injury in mice (17).

Furthermore, Shreesh et al. discovered that pretreatment with TQ reduced AST and ALT in rats with isoproterenol-induced myocardial infarction (28). BE and TQ were also capable of protecting mice from immune-mediated liver injury by reducing the TNF-α's secretion through stimulated -monocytes (14). Previously, it was revealed that TNF-α, an essential inflammatory cytokine, showed a serious function in the etiology of ConA-induced liver injury (7). TNF-α, released by activated T cells and Kupffer cells, could damage hepatic cells by binding to receptors of TNF-α and causing apoptosis in liver cells (3). Both mice treated with TNF-α inhibitors or anti-TNF-α antiserum (29) and mice lacking TNF- receptors demonstrated significant resistance to ConA hepatic injury (8).

As indicated in the current study, the second protective effect of BE and TQ was mediated by a decrease in INF γ expression. IFN-γ was found to be significant in developing ConA liver disease. The significance of IFN-γ might be attributed to its collaboration with TNF-α, which induced the generation of numerous chemokines and adhesion molecules. Furthermore, IFN-γ-induced phosphorylation of STAT1 led to ConA-induced acute liver injury by promoting pro-apoptotic gene expression (30). These findings implied that increasing TNF-α and IFN- γ caused liver injury in the ConA model (7). Therefore, the reversal action of BE and TQ against TNF-α's elevated levels -and IFN-γ indicated the ability of these tested agents to hinder ConA-induced T-cell stimulation and thus alleviate liver injury (23). These results were provided by Jia et al. They showed that BE suppressed TNF-α's mRNA transcription and protein secretion -by macrophages in LPS-induced cytokine expression and inhibited superoxide secretion by neutrophils in a dose-dependent manner (26), also significantly inhibiting TNF-α production in a peritonitis model (31).

Similarly, El-Mahmoudy et al. revealed that TQ reduced TNF-α and thus improved autoimmune Diabetes Mellitus (32, 33). Umar et al. found that the inhibition of TNF-α by TQ in Wistar rats ameliorated collagen-induced arthritis (34). Arslan and Parlar found that TNF-α was reduced after TQ administration for experimental animal models, which reduced the score of autoimmune arthritis (35). Hanieh et al.'s findings were consistent with the current findings. They confirmed that TQ had both immunomodulatory and anti-inflammatory effects. The anti-inflammatory properties were primarily achieved by inhibiting TNF (22). The mechanisms by which BE and TQ reduced ConA-induced TNF-α and IFN-γ expression were then investigated.

The stimulation of the main transcription factor NF-κB is a vital signaling cascade in the upraises of these inflammatory mediators. In this study, administration of BE and TQ to groups III and IV decreased the activation of NF-κB induced by ConA in the intrahepatic inflammatory cells. We therefore postulate that BE and TQ can prevent NF-κB activation in leukocytes found inside hepatic cells, especially T-cells in the liver, and thereby prevent transcription of the target genes, such as various pro-inflammatory cytokines and chemokines, protecting mice from ConA-induced hepatic inflammation and injury (36). IKKα activity may have been suppressed, avoiding the phosphorylation and degradation of IB, which would explain these inhibitory activity (27). In one study, Xin Zhao and colleagues discovered that ConA significantly increased the critical proteins' phosphorylation in the MAPK cascade (ERK, JNK, and p38), while artesunate pretreatment significantly decreased it (37). Based on these findings and analyses, it is reasonable to speculate that inhibiting the stimulation of the NF-κBκκ and MAPK pathways may play a role in the beneficial effect of BE and TQ pre-treatment on Con A-immune mediated liver injuryinjuryinjury. Specific MAPK inhibitors should be used in future research to clarify the TQ and BE actions' exact anti-inflammatory mechanism. These results agreed with LIU Xin- Yu and coworkers, which showed that BE reduced inflammatory processes in hepatic cells induced by ethanol in mice *via* the inhibition of STAT 3, NF- κB, and ERK pathway (38).

Recently, it has been reported that BE has a potential anti-inflammatory effect, which is affected *via* the MAPK and NF-κB signaling pathways' inhibition (26). Several studies showed that BE repressed cancer cell proliferation *via* the NF-κB signaling). According to Akter et al., TQ could potentially treat autoimmune diseases by controlling the NF- signaling pathway. In the multiple sclerosis model induced in rats, the daily administration of TQ alleviated myelin basic protein-induced autoimmune encephalomyelitis by inhibiting the NF- κB activation in the brain and spinal cord (39). In another study, TQ had anti-inflammatory properties, according to Sethi et al., because it inhibited NF-κB and IkBα kinase stimulation. Aside from the MAPK and NF-κB pathways, they regulatedκκ the production of pro-inflammatory cytokines (40). The family of mammalian MAPKs includes p38, JNK, and extracellular signal-regulated kinase (ERK). They are activated by various intracellular and extracellular stimuli, comprising cellular stressors and cytokines. Inhibiting the MAPK pathway could reduce the severity of experimental autoimmune hepatitis. Previous research indicated that NF-κB mightκκ be a p38 MAPK effector. The impairment of p38 MAPK activation might reduce the NF-κBκκ activation, implying that p38 MAPK upregulation promoted the inflammatory cytokines' release in autoimmune liver injury *via* an NF-κBκκ-dependent mechanism (41). Shujin Wu and colleagues discovered that inhibiting the phosphorylation of JNK, a member of the family of mitogen-activated protein kinases, significantly reduced ConA-induced immune hepatitis (42). ConA administration induced JNK phosphorylation, causing liver damage because p-JNK moved from the cytoplasm to the mitochondrial membrane complex or the cell nucleus. ConA stimulated JNK *via* a ROS-dependent mechanism that activated apoptosis signaling kinase 1, blocking hepatic ROS production and almost totally preventing hepatic JNK activation and damage, showing that ROS played a significant role in JNK activation.

Furthermore, oxidative stress played a crucial role in the ConA model, causing DNA damage, inducing apoptosis, and promoting the generation of pro-inflammatory cytokines in hepatocytes (43). According to Forouzanfar and Hosseinzadeh, TQ could scavenge oxidative free radicals such as O_2_, OH, and H_2_O_2_ and be responsible for controlling oxidative stress (44). Other research indicated that BE and TQ contained potent antioxidants capable of scavenging a wide range of ROS, including hydroxyl radicals, superoxide, and peroxide radicals (45, 46). As a result, the third protective effect of BE and TQ could be attributed to their powerful antioxidant effects, which reduced hepatocyte deaths.

## Conclusion

The results demonstrated that both BE and TQ attenuated ConA-induced acute liver injury mainly *via* suppressing the TNF-α and IFN-γ's production and blocking the NF-κB signaling. These findings give insight into developing a new therapeutic agent for treating immune-mediated hepatitis and other viral/autoimmune hepatitis utilizing natural compounds.

## Data availability statement

The raw data supporting the conclusions of this article will be made available by the authors, without undue reservation.

## Ethics statement

The animal study was reviewed and approved by Department of Pharmacology and Toxicology, College of Pharmacy, University of Baghdad, Iraq. Written informed consent was obtained from the owners for the participation of their animals in this study.

## Author contributions

Conceptualization, methodology, and project administration: SK, WA, and MZ. Data curation, writing original manuscript, and review and editing the manuscript: WA. Manuscript preparation: SK and WA. Investigation: SK and MZ. All authors contributed to the article and approved the submitted version.

## Conflicts of interest

The authors declare that the research was conducted in the absence of any commercial or financial relationships that could be construed as a potential conflict of interest.

## Publisher's note

All claims expressed in this article are solely those of the authors and do not necessarily represent those of their affiliated organizations, or those of the publisher, the editors and the reviewers. Any product that may be evaluated in this article, or claim that may be made by its manufacturer, is not guaranteed or endorsed by the publisher.
